# By the Seat of Our Pants: the Experience of Small Businesses in the COVID-19 Pandemic, Washington State, March–October 2020

**DOI:** 10.5888/pcd19.210366

**Published:** 2022-03-24

**Authors:** Peggy A. Hannon, Kristen Hammerback, Christine M. Kava, Perla Bravo-Acevedo, Michelle Strait, Jeffrey R. Harris

**Affiliations:** 1 Department of Health Systems and Population Health, University of Washington, Seattle, Washington

## Abstract

**Introduction:**

Most US businesses are small, yet they employ almost half of the nation’s workforce. Literature is limited about how small employers (those with 20–250 employees) have made decisions about operating their businesses during the COVID-19 pandemic. We sought to learn how employers made these decisions, what information sources they used, what information they wanted, and to what extent they worked with or used information from their local health department.

**Methods:**

We conducted qualitative, semistructured interviews with 26 employers in Washington State, from August through October 2020. Employers were recruited from 7 counties (4 urban and 3 rural) that were experiencing either higher or lower COVID-19 case rates than Washington State overall.

**Results:**

Employers relied heavily on national government resources to make decisions about how to operate their businesses during the COVID-19 pandemic. Few employers had relationships with or turned to their local health departments for information or support. Employers wanted information about COVID-19 safety that was specific to their business operations and industry. Employers also described the emotional toll of COVID-19 and the challenge of trying to make high-stakes decisions with rapidly evolving information.

**Conclusion:**

Small employers showed little awareness of their local health departments and the information and assistance they could provide. Local health departments could increase their visibility and build relationships with small employers by partnering with them on value-added services such as workplace health promotion. Establishing these relationships could support more rapid collaboration between local health departments and small employers during future public health crises.

**Summary**

**What is already known on this topic?**

Many businesses closed because of the COVID-19 pandemic. Relatively little is known about how small businesses made decisions about whether and how to operate in 2020.

**What is added by this report?**

Most small employers we interviewed relied on national government sources for information about the pandemic; most had limited awareness of and no relationship with their local health departments. They desired information that was specific to their industry and felt unprepared for the pandemic.

**What are the implications for public health practice?**

Small employers were open to receiving information from their local health departments. Local health departments may want to increase their partnerships with community employers by providing services that employers value.

## Introduction

Washington State was the site of the first COVID-19 outbreak in the US and thus has had the longest experience with the pandemic. In March 2020 and the ensuing months, substantial restrictions were put in place to limit the spread of COVID-19. Most people, excluding essential workers, were ordered to stay at home for several months, and many businesses either had to close temporarily or find a way for their employees to work from home (1). Several national cross-sectional surveys showed the massive impact of COVID-19 and its necessary restrictions on business operations and revenue (1–3). This impact was especially severe for small businesses (defined by the US Census as having <500 employees), which were more likely to permanently close, lay off staff, and lose revenue (2,3). Small businesses make up 99% of all US businesses and employ almost 50% of US employees (4). They are also more likely to employ workers for low wages and to have a workforce at higher risk for chronic disease (5,6). Thus, employees at small businesses are more likely to be at risk for income and health disparities, and these risks are exacerbated by COVID-19 (7).

At this writing, little research is available on how employers that operate small businesses made critical decisions early in the pandemic. These decisions included whether to remain open and how to operate, which information sources to trust and use to guide operating decisions, and how to engage and communicate with employees about these decisions. Local health departments possess local information about COVID-19’s spread and its risk in a given community and also have access to support and guidance from the Centers for Disease Control and Prevention (CDC) and state health departments. For this reason, they seem like a natural information source and partner for small businesses as they try to keep abreast of the status of COVID-19 in their community and make decisions about the safety of their employees and their customers (8). Local health departments also want to partner with more businesses in their communities on other initiatives, such as workplace health promotion and emergency response initiatives, to improve community health and increase their reach into the community (9–12). However, these partnerships are subject to potential barriers. Employers that have relationships based on regulatory compliance may view local health departments in an adversarial light; other employers may have limited awareness of their local health department and how it can provide assistance related to employee health and well-being (10). However, several examples exist of these departments successfully partnering with businesses in their communities to address local health crises such as homelessness, infant mortality, and food insecurity (12).

Our goal was to learn how small employers made decisions related to the COVID-19 pandemic, the types and sources of information they used to inform their decisions, who made the decisions, and the role of local health departments in providing information or other assistance. This information may be useful to health departments in assisting small businesses in dealing with the pandemic; such information may also help them partner and effectively share resources with small businesses in future public health emergencies and could inform their health promotion and other partnership efforts (13). In this article, we use “employer” when talking about an owner or decision maker, “business” when describing a firm or enterprise, and “worksite” when describing the physical location of a business.

## Methods

### Setting

We interviewed 26 employers from 7 counties in Washington State that represented a mix of urban and rural populations, as defined by the Washington State Department of Health, (www.doh.wa.gov/Portals/1/Documents/Pubs/609003.pdf). Counties were also selected on the basis of population size and the degree of the pandemic impact, defined as the case rate per 100,000 population for March 1 through June 10, 2020. Counties were defined as either experiencing lower rates of COVID-19 infections (fewer than 100 cases per 100,000) or higher rates (more than 350 cases per 100,000) compared with Washington State as a whole, which had a case rate of 341 per 100,000. We obtained case-rate data from the Washington State Department of Health’s COVID-19 Data Dashboard (from summary tables for case rates, by county) (14). We used pandemic impact to select counties to determine whether employers made different decisions or used different decision-making processes on the basis of COVID-19 prevalence in their communities.

### Eligibility

The following were eligibility criteria for participation in our study:

1. The worksite was located in one of the following 7 counties: Benton, Cowlitz, Franklin, King, Kitsap, Lewis, or Snohomish. Benton and Franklin counties share a local health department.
2. The business had 20 to 250 employees. We selected this subset of businesses within the small employer definition of <500 employees because it is consistent with our other work with small employers and local health departments (15,16).
3. The business’s industry category was one of the following: accommodation and food services, health care, social assistance/nonprofit organization, retail trade, and other services, excluding public administration. We chose these 5 categories because they pay disproportionately low wages compared with the industry average for Washington State and employ more than 50 million people nationwide (17).
4. The business continued to operate at some level during the pandemic at the time of the interview, including remote work. Businesses that were closed during the recruitment period were excluded, in part because they were difficult to reach and in part because we wanted to talk with employers who were making decisions about how to keep their businesses running and maintain employee safety.
5. The business had a representative who was able to answers questions about how the business had responded to the COVID-19 pandemic. Questions included how COVID-19 and social distancing measures affected their operations, how decisions were made during the pandemic about steps related to employee health and safety and whether to remain open, and what resources were used to guide the decision-making process. Representatives did not have to serve as primary decision maker, but they needed to know how and why key decisions were made.

### Recruitment

All employers were recruited by Focus Insite (focusinsite.com), a commercial market research firm based in West Chester, Pennsylvania. Focus Insite used a purchased list to identify and call potentially eligible businesses in the 7 counties. The name (first name, last initial) and contact information of eligible and willing representatives of selected businesses were emailed each day to a researcher (K.H.), who then contacted the representative via email or telephone to arrange an interview time. Respondents were offered an incentive of $75 to participate.

### Measures and procedures

Three researchers (K.H., C.M.K., P.B.-A.) used a semistructured guide to conduct interviews via teleconference or telephone. Questions included the effects of COVID-19 on their business’s decisions about employee health and safety, whether and how to stay open, sources of information used and preferred to guide decision making, and the role of their local health department in those decisions. Respondents were also asked what they wished they had known before the COVID-19 pandemic about maintaining the health and well-being of their employees and how their local health department might prove useful in providing information and support with future pandemic or pandemic-type events. Interviews lasted approximately 30 minutes and were recorded with respondents’ consent. Interviews were professionally transcribed by Focus Insite. The University of Washington Institutional Review Board declared the study exempt from review.

### Analysis strategy

The research team developed a coding structure based on reviews of the transcript, the interview guide, and primary research questions. Researchers K.H., C.M.K., and P.B-A. used Atlas.ti, version 8 (18) to code transcripts. To ensure consistency in coding, we first double-coded a portion of the transcripts and then met to discuss disagreements in coding. The coding structure was subsequently refined and used by the researchers to independently code all transcripts. We generated code reports in Atlas.ti to examine patterns in the data to identify key themes.

## Results

Twenty-six employers from 7 counties completed interviews (Table).

**Table:** Characteristics of Employers and Small Businesses Surveyed (N = 26), 7 Counties in Washington State, March–October 2020

Characteristic	N
**Age, y**	
18–39	12
40–54	9
≥55	5
**Sex**	
Female	16
Male	10
**Job title**	
Human resources manager	8
Operations manager	5
Owner	2
Sales/customer service manager	5
Other	6
**No. of employees**	
<100	17
100–250	9
**County**	
Benton	1
Cowlitz	2
Franklin	3
King	10
Kitsap	2
Lewis	2
Snohomish	6
**Rural/urban**	
Rural	10
Urban	16
**Industry**	
Accommodation and food services	6
Health Care	6
Other services	9
Retail	3
Social assistance/nonprofit	2
**COVID-19 case rate**	
High	20
Low	6

### Decision-making process during COVID-19

All employers recognized the potential impact of COVID-19 on their worksite operations and employee health, and they reported their goal of reducing COVID-19 transmission at their worksites. In general, they did not feel prepared to deal with a pandemic and did not report a pre-established process for making decisions about how to manage their business during a pandemic. “ It was kind of fly by the seat of our pants because everything was changing so rapidly.” Because these were small businesses in low-wage industries, most did not have any workplace health promotion programs in place at the start of the pandemic.

For the most part, leadership and managers made the decisions about how to conduct business during the pandemic. Decisions about whether and how to remain open were often guided by whether the company could survive a temporary shutdown, whether the company was an essential business, and the COVID-19 case rate in their community. Several sought some employee input during the decision-making process. Most employers used digital communications, such as email, to convey these decisions to their employees. “Yes, it was ultimately myself, the HR manager, taking the information directly to the owner and talking through the recommendation, why I felt it was necessary, any regulations that may have an impact like the stay-at-home order, those sorts of things. . . . I would generally get their buy-in and I would send employee email letting them know, ‘This is now how we’re doing things,’ or, ‘This is now an extra safety measure that we’ve made,’ or something like that.”

### Information sources

Employers used various information sources to guide their decisions. The most commonly reported information sources were CDC, the World Health Organization, Washington Governor Jay Inslee’s website, and the Washington State Department of Health’s website. Most small employers in our study trusted these sources. “The CDC, the Employment Security Department, the Governor’s website . . . those are sites I tend to trust most.” Only 2 employers said that they were not sure they could trust government information or were unsure of the need for measures like stay-at-home orders. A few employers used resources from the Society for Human Resource Management that were specific to their industry (employers were particularly interested in guidance specific to their industry and circumstances).

Most employers had no relationship with their local health department and did not seek COVID-19 information from them. “They’re just not on my radar, but I’d go to them if they had something to offer.” The few that did report using information from local health departments or calling them had already established relationships before COVID-19. These relationships usually were either regulatory in nature (that is, the local health departments ensured they complied with mandated practices) or were a partnership (for example, one respondent collaborated with a city that worked closely with their local health department on several issues). In a few instances, employers said they were reluctant to go to their local health department because of previous negative experiences, but lack of awareness of, or relationship with, local health departments was far more common. Employers were open to receiving information from their local health departments but were generally interested only if this information was new or different from what they already had access to.

### Desired information and communication channels

The most common message from employers was that they wanted industry-specific information about managing their business during COVID-19. We heard this both from employers that were in industries that were receiving industry-specific guidance and from those that were not. Some discussed either looking to their peer businesses in their industry to see what they were doing or said they wanted someone to convene groups of businesses similar to theirs to discuss best practices. This theme also came up when we discussed their relationships with and desire for information from their local health departments. Businesses wanted industry-specific information that, ideally, also accounted for the local context. “If it were really pointed information that was industry-specific, as opposed to just a generalization, and if it were less along the just wear your mask, stay 6 feet apart. If it really provided something in order to move into another phase and took into account where we’re located, or how to resume our types of activities, that this is what we can do as businesses to really, truly keep our employees safe . . . well that might be helpful, but other than that, I think no.”

Employers who were interested in receiving information from their local health departments expressed interest in various topics, including

- How to keep employees motivated and engaged during the pandemic and while working from home
- Reliable information about how COVID-19 was affecting their community and about local containment guidelines
- How to communicate information about the COVID-19 vaccine (once the vaccine was available)
- How to get information tailored to their industry
- Mental health resources for employees
- Other resources to support employees, such as information about affordable housing

Most participants preferred email or internet-based communication channels.

### Emotional toll of COVID-19

Several employers made comments related to the emotional toll of trying to manage their business and support their employees during the pandemic. These comments addressed the stress of not knowing what to do: “It’s hard when you're in that type of role to throw your hands up and say, ‘I know pretty much as much as you do because I’ve never dealt with a pandemic in my lifetime before.’”; “I think what's been most frustrating in that is just the different perspectives from the different agencies on what is required or needed.” Another comment included guilt about having to lay off or furlough employees: “We ultimately had to furlough 18 employees, but if we’d known how long this would last we would have encouraged them to find new jobs and I feel so bad about that.”

When we asked employers what they wished they had known before the pandemic, many found this question hard to answer. Some said the pandemic made them realize how ill-prepared they were to handle a pandemic or emergencies. Others wished they had known more about COVID-19 symptoms, mode of transmission, and preventive strategies in the beginning or more quickly.

## Discussion

Our interviews showed that small employers felt unprepared for the unexpected health crisis that the COVID-19 pandemic created and were emotionally taxed by it. Although they viewed global, national, and state government sources of information as trustworthy and useful, they were unfamiliar with their local health department and did not look to it for information. Perhaps because our study was conducted relatively early in the pandemic, we did not detect the mistrust of public health officials, including local health departments, or backlash against them (19) that occurred later as lockdowns wore on and were accompanied by mask and vaccine mandates.

Our findings align with those of others in terms of lack of certainty about best practices and their duration and the impact of financial considerations on decisions about whether to remain open (2). Our study is unique because of its focus on operations during the pandemic and on employee health and safety. Our findings also align with a New York survey in which local health department directors rated their organizations as least effective in their coordination with local businesses in the domains of public health preparedness systems and communications (20). Our findings suggest several ways that local health departments could connect with small employers and help them during future pandemics or other health crises. In our study, employers welcomed materials on how to operate relatively safely during the pandemic; local health departments could develop or curate and distribute these materials. Employers indicated that industry-specific guidance would be particularly helpful, although local health departments might need to collaborate with other partners to produce these materials. Convening peer employers could also serve to collect and disseminate local best practices on an industry-specific basis (we worked with 2 local health departments in the past who successfully brought together local employers to discuss workplace health promotion).

Local health departments can face challenges in their relationships with employers. We found that the departments are often unknown to small employers, and when they are known, the health protection they offer is often regulatory (eg, inspections of restaurant and food processing facilities, environmental protection, licensing of businesses such as day-care centers and campgrounds) (21). Thus, some employers may view the local health department as threatening. In addition to partnering on pandemic preparedness, local health departments could build and improve their relationships with small employers (8,13) by providing other nonregulatory services likely to be viewed as positive, such as workplace health promotion. A recent study supported by the CDC Foundation (Vandiver Group report on CDC Foundation focus groups with members of the National Association of County and City Health Officials) found that local health departments are highly interested in partnering with employers on workplace health promotion. CDC’s Workplace Health Resource Center offers ample tools and resources, and local health departments could provide the technical assistance and support that small employers have been shown to need to implement these programs (22). They can also work with employers via community partners that already have strong relationships, such as local chambers of commerce, Rotary clubs, and other community-based organizations. However, because local health departments vary in size and capacity, most need funding aligned with employer partnerships to take on such partnership activities (9).

Our study has several limitations. We limited our sample to employers from 7 counties in Washington State. Washington has fared well during the pandemic, with the seventh lowest death rate among the 50 states, the District of Columbia, and Puerto Rico (23). Therefore, the perspectives presented here may differ from those of other states. We also interviewed only people who spoke English. Though we included employers that were operating remotely, we spoke only to those that remained open during the interview period. Thus, we did not learn about the decision-making processes of businesses that decided to close operations during the pandemic. Because our interviews were conducted before vaccines were available, we collected no information related to COVID-19 vaccinations.

Our study also has strengths. Our sample included a variety of industries and employers in both urban and rural areas, yet we achieved saturation. On the basis of our findings, we can also offer specific recommendations for what might help in the event of another crisis like COVID-19 and in preparation for such a crisis.

Small employers in Washington State were deeply affected by the pandemic and struggled to find tailored information on how to operate their workplaces while protecting their employees. They welcomed information from government sources but showed little awareness of their local health departments and the information and assistance they could provide. In future health-related crises like the COVID-19 pandemic, local health departments have an opportunity to provide small employers with information and other support to operate safely. In the meantime, local health departments may want to increase their visibility and build relationships with small employers by partnering with them on value-added services such as workplace health promotion.
